# Identification and Characterization of MicroRNAs Controlled by the Osteoblast-Specific Transcription Factor Osterix

**DOI:** 10.1371/journal.pone.0058104

**Published:** 2013-03-05

**Authors:** Qin Chen, Wenbin Liu, Krishna M. Sinha, Hideyo Yasuda, Benoit de Crombrugghe

**Affiliations:** 1 Department of Genetics, The University of Texas MD Anderson Cancer Center, Houston, Texas, United States of America; 2 Department of Bioinformatics & Computational Biology, The University of Texas MD Anderson Cancer Center, Houston, Texas, United States of America; 3 Department of Endocrine Neoplasia and Hormonal Disorders, The University of Texas MD Anderson Cancer Center, Houston, Texas, United States of America; Georgia Regents University, United States of America

## Abstract

Osterix (Osx) is an osteoblast-specific transcription factor which is essential for bone formation. MicroRNAs (miRNAs) have been previously shown to be involved in osteogenesis. However, it is unclear whether Osx is involved in the regulation of miRNA expression. In this study, we have identified groups of miRNAs that are differentially expressed in calvaria of the E18.5 *Osx^−/−^* embryos compared to wild type embryos. The correlation between the levels of miRNAs and Osx expression was further verified in cultured M-Osx cells in which over-expression of Osx is inducible. Our results suggest that Osx down-regulates expression of a group of miRNAs including mir-133a and -204/211, but up-regulates expression of another group of miRNAs such as mir-141/200a. Mir-133a and -204/211 are known to target the master osteogenic transcription factor Runx2. Further assays suggest that *Sost*, which encodes the Wnt signaling antagonist Sclerostin, and alkaline phosphatase (*ALP*) are two additional targets of mir-204/211. Mir-141/200a has been known to target the transcription factor *Dlx5*. Thus, we postulate that during the process of Osx-controlled osteogenesis, Osx has the ability to coordinately modulate Runx2, Sclerostin, ALP and Dlx5 proteins at levels appropriate for optimal osteoblast differentiation and function, at least in part, through regulation of specific miRNAs. Our study shows a tight correlation between Osx and the miRNAs involved in bone formation, and provides new information about molecular mechanisms of Osx-controlled osteogenesis.

## Introduction

Osteoblasts arise from mesenchymal precursors and play critical roles in osteogenesis. The progression through osteoblastic commitment, proliferation, and terminal differentiation integrates diverse signals and expression of differentiation marker genes. The marker gene expression and osteoblast differentiation are governed by expression of several transcription factors, especially Osterix (Osx), runt-related transcription factor 2 (Runx2), and distal-less homeobox 5 (Dlx5) [1], [2], [3], [4], [5], [6]. In addition, several microRNAs (miRNAs) have recently been discovered as important regulators of osteoblast gene expression [7], [8], [9], [10].

Osx is a C2H2-type zinc finger-containing transcription factor that is specifically expressed in osteoblast lineage cells, and is essential for osteoblastogenesis and bone formation. In mice with deletion of the *Osx* gene (*Osx^−/−^*), formation of both endochondral and intramembranous bone was completely abolished [1], [2]. Primary culture of calvarial osteoblasts of the *Osx^−/−^* mice showed increased BrdU incorporation, indicating that those osteoprogenitors were less able to exit their cell cycle progression without Osx [11]. The normal progression of osteoblast differentiation is accompanied by expression of several markers, such as type I collagen (COL1), ALP, bone sialoprotein (BSP), and Osteocalcin (OC) [1]. In *Osx^−/−^* mice, since differentiation of osteoblasts was arrested, expression of these markers was dramatically decreased [1], [2], [12], highlighting an essential role of Osx in the differentiation of osteoprogenitors into mature and functional osteoblasts. Moreover, our in vitro study has recently shown that Osx can bind to and transactivate *Bsp* as well as *Oc* promoters [13]. It is not yet clear whether Osx interacts with the *ALP* gene. In addition, our previous study showed that expression of *Runx2* mRNAs was increased in calvaria of the *Osx^−/−^* mice [14].

Runx2 is a critical regulator of the osteogenic lineage. *Runx2^−/−^* mice did not form osteoblasts and failed to express osteoblastic differentiation markers [3], [4]. Similar to Osx, Runx2 can also bind to and regulate expression of these marker genes [15]. Since expression of *Osx* mRNA in the *Runx2^−/−^* mice was undetectable, whereas expression of *Runx2* mRNA in the *Osx^−/−^* mice was increased, it suggests that Runx2 acts upstream of Osx. In fact, Runx2 can directly bind to *Osx* gene and transactivate its promoter activity [16]. Presently, little is known about whether Osx regulates the level of Runx2 protein. Interestingly, in the *Runx2* gene P1 promoter region, certain Dlx5-responsive elements have been identified [17]. Dlx5 is another transcriptional regulator of osteogenesis. Unlike the *Osx^−/−^* or *Runx2^−/−^* mice, *Dlx5^−/−^* mice showed less severe bone abnormalities [6], indicating that Dlx5 is not essential in osteoblastogenesis. Nevertheless, when over-expressed in chicken calvarial cells, Dlx5 accelerates osteoblast differentiation [18], suggesting that maintenance of an optimal level of Dlx5 is important for formation of normal bone tissue. So far, it is not clear whether Osx regulates Dlx5 expression. Expression of *Osx*, *Runx2* and *Dlx5* is regulated by a broad signaling network including members of the Wnt family. The canonical Wnt/β-catenin signaling can promote osteoblastic fate determination, proliferation and survival [19], [20], [21]. A previous study showed that the interaction of Wnt ligands with Lrp5/6 co-receptors can be antagonized by Sclerostin (encoded by *Sost* gene) [22]. In *Sost^−/−^* mice, Wnt signaling and bone mass was increased [23], whereas transgenic mice over-expressing *Sost* in bone tissue had osteopenia [24], indicating that Sclerostin is an important negative regulator of bone formation. It has been known that Osx can directly bind to the *Sost* gene and transactivate its promoter activity [14], [25]. However, whether the control of *Sost* expression by Osx also involves miRNAs has not yet been examined.

MiRNAs, a form of non-coding RNAs (ncRNAs), have recently emerged as important regulators in diverse cell proliferation and differentiation processes. These endogenous ncRNAs are single-stranded small RNA molecules that consist of ∼22 nucleotides and are evolutionarily conserved [26]. They regulate protein translation or mRNA stability by binding to the 3′-UTR of their target genes. Since miRNAs can bind to more than one target, it has been proposed that they regulate up to 30 percent of the protein-coding genes in the genome, highlighting their importance as regulators of gene expression. Several in vitro cell culture studies reported that many miRNAs, such as mir-23a, -30C, -34C, -93, -133, -137, -141/−200a, -204/−211, -2861 and -3960 are involved in osteogenesis [8], [9]–[10], [27], [28], [29], [30]. In particular, expression of mir-141 or its homologue -200a can repress BMP2-induced preosteoblast differentiation by the translational suppression of Dlx5 [28]. Mir-93 has been recently found to directly target Osx and inhibit osteoblast mineralization [10]. Mir-133, −204 and its homologue −211, were previously identified as Runx2-targeting miRNAs [27], [29]. Recently, a panel of 11 miRNAs, including mir-133a and -204, have been found to be expressed in a lineage-related pattern in mesenchymal cell types, and to directly target the 3′-UTR of the *Runx2* gene [30]. When over-expressed in cultured MC3T3 cells, all of these miRNAs inhibited osteoblast differentiation as well as ALP production [30]. Moreover, a knockout study showed that conditional deletion of *Dicer* in cells of osteoblast lineage, which blocks formation of mature miRNAs, causes overt abnormality of bone formation [31], demonstrating a critical role of miRNAs in the regulation of osteoblast differentiation and bone formation.

To date, the physiological role of miRNAs in the regulation of osteoblast differentiation in vivo has not been well defined. In particular, we do not know whether deletion of the *Osx* gene, which results in a block in osteoblast differentiation and bone formation, causes changes in expression of specific miRNAs. To address this question, we first used miRNA array hybridizations comparing miRNA expression profiles between *Osx^−/−^* and wild-type calvaria of mouse embryos at E18.5. To identify the Osx-regulated miRNAs, we then used an inducible Osx-expressing cell line to further validate expression of several differentially expressed miRNAs with qPCR assays. Finally, using transfection assays we tested whether expression of selected miRNAs would interfere with osteoblast differentiation in vitro, and whether mir-204/211 was able to directly target the 3′-UTR of *ALP* or *Sost* gene and attenuate their protein synthesis.

## Materials and Methods

### Ethics Statement

All experimental procedures described in this study were approved by the Institutional Animal Care and Use Committee of The University of Texas MD Anderson Cancer Center (IACUC Protocol No: 108807638).

### Animals

Homozygous *Osx^−/−^* mice were generated and described previously [2]. Genotyping was performed as described previously [2].

### Total RNA Isolation

The total RNAs of cultured cells and RNAs of calvaria from E18.5 wild type or *Osx* ^−/−^ mouse embryos were isolated using TRIzol reagent (Invitrogen, 15596-018) [32]. The RNA quality control was performed using a Bioanalyzer 2100 (Agilent).

### MiRNA Array, Data Collection and Analysis

MiRNA microarray processes, including probe library construction, printing, labeling, hybridization, image scanning, and initial data analysis, were conducted by the Center for Targeted Therapy (the Sequencing and Non-coding RNA Program) at The University of Texas MD Anderson Cancer Center, following an miRNA profiling protocol as described previously [32]. A 15K human/mouse miRNA/ncRNA chip was used. Within this chip, the mouse miRNA oligo probes were designed and derived from 720 *Mus musculus* miRNAs in the Sanger miRBase database (http://microrna.sanger.ac.uk/cgi-bin/sequences/browse.pl). The raw signal intensity data were generated using the software GenePix Pro 6.1 on the Axon 4000B scanner (Molecular Devices). The background corrected raw data (the “F635 Median - B635” column in the.gpr files) were checked for quality and filtered as follows: Buffers and blank spots were filtered first; the miRNA that signal intensity was low (i.e., a flag −50 in the “Flags” column) in at least five out of the six samples was excluded from further analysis; the low-intensity spots above 50 were assigned a small positive value 1; the replicate spots were averaged. After log2-transformation, the raw data were normalized by quantile normalization [33] implemented in the R package *limma* [34]–[35], [36]. Differential expression was detected using *limma*. At a false discovery rate (FDR) of 5, 10, or 20%, none of the miRNAs showed significant changes in expression between wild type and *Osx^−/−^* sample groups by either Benjamini-Hochberg method [37] or q-value [38], [39]. MiRNAs with raw p value <0.1 and fold-change value of at least 1.5 in either direction were listed as candidate hits for experimental validation (Tables S1 and S2).

### Real-time RT-PCR Quantification of miRNAs

To confirm the microarray results, expression levels of the selected miRNAs were quantified by real-time RT-PCR (qPCR) using the miScript PCR System (QIAGEN) according to vendor’s instructions. In brief, miRNAs were polyadenylated by poly (A) polymerase and subsequently converted into cDNA using reverse transcriptase with oligo (dT) priming. Reverse transcription reaction contained 1 µg of RNA template, 1 µl of miScript Reverse Transcriptase Mix, and 4 µl of miScript RT Buffer, which includes Mg2+, dNTPs, and primers. Reaction mixture (20 µl) was incubated for 60 min at 37°C, then 5 min at 95°C. QPCR was performed using an Applied Biosystems 7500 sequence detection system; the reaction mixtures (50 µl) included 5 µl of cDNA template, 25 µl of SYBR Green PCR Master Mix, 5 µl of miScript Universal Primer, and 5 µl of miScript Primer Assay. Reactions were distributed into a 96-well optical plate and incubated at 95°C for 15 min in the real-time cycler, followed by 40 cycles of 94°C for 15 s, 55°C for 30 s, and 70°C for 30 s. The threshold cycle (Ct) was adjusted from a default “Manual Ct” threshold value of 0.2 to a lower value of 0.02. The Ct value was defined as the fractional cycle number at which the fluorescence passed the fixed threshold. U6 small RNA was used as an internal control to normalize the miRNA input. The sequences for miScript Primer Assay are shown in Table S3. For each selected miRNA, the qPCR experiment was performed in triplicate with three independent batches of cDNAs. Changes (x-fold) in miRNA expression level were calculated by the equation 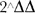 Ct, where ΔΔCt = (Ct, Target – Ct, U6) Target sample – (Ct, Control – Ct, U6) Control sample [40]. All statistical analyses were performed with two-tailed Student’s t tests using Microsoft Excel software. Results are the means ± S.D. Data were considered to be significantly different for p<0.05.

### Generation and Culture of M-Osx Cells

M-Osx cells have been generated by stably expressing inducible Osx expression plasmid vectors in mouse preosteoblast MC3T3 cells. Briefly, MC3T3 cells were first stably transfected with pTet-off (Clontech). The positive clones selected by G418 were then stably transfected with pTRE-Flag-HA-Osx and pTK-hyg plasmids [13]. After G418 and hygromycin selection, the positive clones were expanded and maintained in the presence of tetracycline (Tet) [13]. Osx expression was turned on or off by absence or presence of Tet in culture media. M-Osx cells were cultured in *α*-minimal essential medium (*α*-MEM) containing 10% fetal bovine serum (FBS) in the absence or presence of Tet. After 48 hours (48 h), cells were harvested for total RNA isolation using TRIzol reagent.

### Generation and Culture of UMR-conRNA and UMR-mir204 Cells

UMR-conRNA and UMR-mir204 cells have been generated by stably expressing inducible expression plasmids of control RNA or mir-204 in rat osteogenic UMR-106 cells. In brief, precursor of mir-204 was amplified by PCR using primers 5′-gctacagtccttcttcatgtg/5′-gttatgggctcaatgatgg selected from intron 6 of *Trpm3* gene. BAC clone R23-473O13 (BACPAC Resources Center) was used as the PCR template. The PCR fragment (∼188-bp) was then inserted into the Xho1/EcoR1 site of pTRIPZ vector (Thermo Scientific Open Biosystems) to generate a mir-204-pTRIPZ plasmid. Non-silencing shRNA-pTRIPZ (conRNA-pTRIPZ) construct was obtained from Thermo Scientific Open Biosystems. The pTRIPZ vector has been engineered to be Tet-On and produce inducible expression of shRNAmir in the presence of doxycycline (Dox). It also contains a puromycin drug resistance gene for selecting stable cell lines, and a RFP (Red Fluorescence Protein) marker for tracking shRNAmir expression. Mir-204- or conRNA-pTRIPZ construct was transfected into the UMR-106 cells by use of Lipofectamine 2000 (Invitrogen) according to manufacturer’s protocol. The cells that incorporate conRNA- or mir-204-pTRIPZ construct were selected by puromycin treatment to generate two stable cell lines, which were named UMR-conRNA and UMR-mir204. When these cells are treated with Dox, expression of mir-204 or control RNA triggers expression of RFP, which shows red color under fluorescence microscope.

### Western Blot

Western blot was performed as described previously [13]. For characterization of M-Osx cells, cells selected from three clones (number 24, 34 and 35) were cultured in *α*-MEM containing 10% FBS in the absence or presence of Tet. For characterization of UMR-conRNA and UMR-mir204 cells, 1.5 × 10^5^ UMR-conRNA or UMR-mir204 cells were cultured in 6-well plate containing DMEM supplemented with 10% FBS in the presence or absence of 1.0 µg/ml Dox. After 40 h (for M-Osx) or 54 h (for UMR-conRNA or UMR-mir204) incubation, cells were collected and suspended with buffer containing 50 mM Tris-Cl (pH 8), 150 mM NaCl, and 1% NP-40 along with protease inhibitor cocktail, and further lysed by 1 × SDS sample buffer. The total proteins in cell lysates were separated on SDS–PAGE, transferred to a nitrocellulose membrane and immunoblotted using anti-Flag (Millipore, MA), anti-Runx2 (MBL international), anti-Osx (Ambcan), or anti-Sost (R&D) antibody, respectively, followed by reaction with appropriate HRP-labeled secondary antibody. The signals were then detected by Super Signal chemiluminescence reagent (Pierce).

### Northern Blot

After G418 and hygromycin selection, cells from the positive clone (number 35) were grown in *α*-MEM containing 10% FBS, Tet, and in the presence or absence of BMP2. After 40 h, cells were collected for total RNA isolation using TRIzol reagent. Northern blot was performed as previously described [41]. In brief, total RNA (15 µg) was electrophoresed in a 1.2% agarose-formaldehyde gel, transferred onto Hybond-N-membrane (Amersham International, Amersham, UK), and hybridized with (α-^32^P-dCTP)-labeled cDNA probes overnight at 42°C. After serial washing with 6 × and 2 × Standard Saline Citrate (SSC) plus 0.1% SDS, membranes were developed by auto-radiography. The cDNA probes used in this study are: mouse *Osx* cDNA containing 5′ fragment, mouse *Runx2*, mouse *Bsp,* and mouse *Oc*.

### Reporter Constructs

Fragments containing different regions of the *Sost* 3′-UTR were generated by PCR amplification and were cloned into the *Hin*d III/*Spe* I sites at the 3′ polylinker of pMIR-REPORT, a miRNA expression vector (Ambion). It contains a CMV promoter that directs expression of the firefly luciferase gene and a 3′-polylinker, which allows insertion of 3′-UTRs with miRNA seed sequences, followed by a SV40-derived polyadenylation site. Two ∼200-bp fragments spanning either a proximal (pSost1) or a distal (pSost2) 3′-UTR region, and one ∼700-bp fragment containing both proximal and distal 3′-UTR regions of *Sost* mRNA (pSost3) were inserted into the pMIR-REPORT vector to generate three reporters for miRNA luciferase assay. All three reporters (pSost1, pSost2, and pSost3) contain either the full mir-204 seed region (pSost3) or clusters of the mir-204 seed regions (pSost1 and pSost2). The primers used for PCR amplification are available on request. Each reporter construct was verified for correction by sequencing before use. For luciferase assay on mir-204 targeting *ALP*, a wild type (p-ALP) and a mutant (p-mALP) ALP reporter were generated. The oligonucleotides 5′-CTAGTAATTTCTCTTTTTGGTGTTGGTTAAAAGGGAACACAAAGACATTTAAATAAA and 5′-AGCTTTTATTTAAATGTCTTGTGTTCCCTTTTAACCAACACCAAAAAGAGAAATTA were annealed, followed by phosphorylation with T4 polynucleotide kinase, and then inserted in the *Hin*d III/*Spe* I site of pMIR-REPORT to produce the p-ALP reporter. The underlined nucleotides are the mir-204 seed sequence (UUCCCUUU). Mutagenesis of this fragment was achieved by annealing the oligonucleotides 5′- CTAGTAATTTCTCTTTTTGGTGTTGGTTATCTCATTACACAAAGACATTTAAATAAA and 5′- AGCTTTTATTTAAATGTCTTGTGAATGAGATAACCAACACCAAAAAGAGAAATTA. After T4 phosphorylation, the mutant fragment was inserted in the *Hin*d III/*Spe* I site of pMIR-REPORT to generate the p-mALP reporter. In the mutated *ALP* fragment, the bases of mir-204 seed sequence AAAGGGA were replaced by TCTCATT to abolish the interaction between a specific binding site in the 3′-UTR and mir-204.

### Transfection Assays

For an ALP activity assay, C2C12 cells were cultured in *α*-MEM supplemented with 10% FBS, 300 ng/ml of BMP2, 50 µg/ml of ascorbic acid, 10 mM β-glycerophosphate. After overnight incubation at 37°C, 50 nM control RNA, mir-204, -302a or -544 was transfected into cells with Lipofectamine 2000 (Invitrogen) following the manufacturer’s protocol. Transfections were done in triplicate. At 72 h after transfection, cells were harvested to assess ALP production using an ALP staining kit (Sigma) following the manufacturer’s instructions.

For miRNA target gene reporter assays, 293T or MC3T3 cells were cultured in Dulbecco’s modified Eagle’s medium (DMEM) or *α*-MEM supplemented with 10% FBS overnight at 37°C before transfection. For transient transfections, 50 ng of an empty pMIR-REPORT vector (empty vector) or a target gene-reporter plasmid was co-transfected with 25 ng of SV40-βgal control plasmid plus or minus mir-204 mimic or anti-mir-204 RNA oligos (50 nM) per well of a 24-well tissue culture plate unless otherwise indicated. Using Lipofectamine 2000 (Invitrogen), the DNA or DNA/RNA mixtures were prepared and added to the cells containing 0.5 ml of fresh DMEM or *α*-MEM supplemented with 10% fetal calf serum. After 48 h incubation, the cells were harvested and assayed for luciferase activity using a luciferase assay device (Promega). The β-gal activity was assayed using the Tropix β-galactosidase kit according to the manufacturer’s instructions (Tropix). Luciferase expression was normalized to the β-gal control. Transfections were done in triplicate, and results were calculated as means ± S.D. from three individual experiments. All statistical analyses were performed with two-tailed Student’s *t* tests. Data were considered to be significantly different for *p*<0.05.

## Results

### MiRNA Array Profiling in Osx-null Calvaria

To establish a correlation between Osx and miRNA expression, we conducted miRNA expression profiling by miRNA-microarray analysis using total RNA isolated from calvaria of E18.5 wild-type (WT) and *Osx^−/−^* mouse embryos. After background reduction and quantile normalization, we found that 40 mouse miRNAs had a 1.5-fold or greater change in expression in the *Osx^−/−^* mutants compared with their WT littermates. Among these miRNAs, 30 had increased expression and 10 had decreased expression in *Osx^−/−^* calvaria relative to WT controls. Among the 30 up-regulated miRNAs, 18 have a 2-fold or greater elevation in their expression level in the *Osx^−/−^* calvaria and are listed in Table S1. The 10 down-regulated miRNAs are shown in Table S2. The changes in expression of these miRNAs are also shown in a heat map (Figure S1). About 60% of miRNAs listed in Tables S1 and S2 were verified by qPCR using total RNAs from calvaria of the *Osx* ^−/−^ and WT mouse embryos at E18.5. MiRNAs with a 3-fold or greater change in their expression level are shown in Tables 1 and 2.

**Table 1:** Down-regulated miRNAs in E18.5 *Osx^−/−^* calvaria validated by qPCR.

Name	Wt (dCt)	Osx−/−(dCt)	p-Value	Ratio(Osx−/− vs Wt)
mmu-mir-141	5.67±0.31	7.34±0.39	0.01	-3.14
mmu-mir-192	6.87±0.26	8.46±0.33	0.01	-3.01
mmu-mir-200a	7.89±0.65	9.49±0.67	0.01	-3.03
mmu-mir-1194	6.66±0.39	8.69±0.58	0.01	-4.08

**Table 2:** Up-regulated miRNAs in E18.5 *Osx^−/−^* calvaria validated by qPCR.

Name	Wt (dCt)	Osx−/−(dCt)	p-Value	Ratio(Osx−/− vs Wt)
mmu-miR-133a	4.50±0.25	2.85±0.24	0.01	3.14
mmu-miR-204	6.11±0.35	4.03±0.34	0.01	4.23
mmu-miR-211	6.29±0.23	4.63±0.45	0.01	3.16
mmu-miR-302a	9.21±0.59	7.25±0.39	0.01	3.89
mmu-miR-433	5.54±0.86	3.14±0.50	0.01	5.27
mmu-miR-501	6.57±0.31	4.98±0.38	0.01	3.01
mmu-miR-544	8.91±0.45	7.09±0.41	0.01	3.51

### Generation and Characterization of M-Osx Cell Line

**Figure 1:**
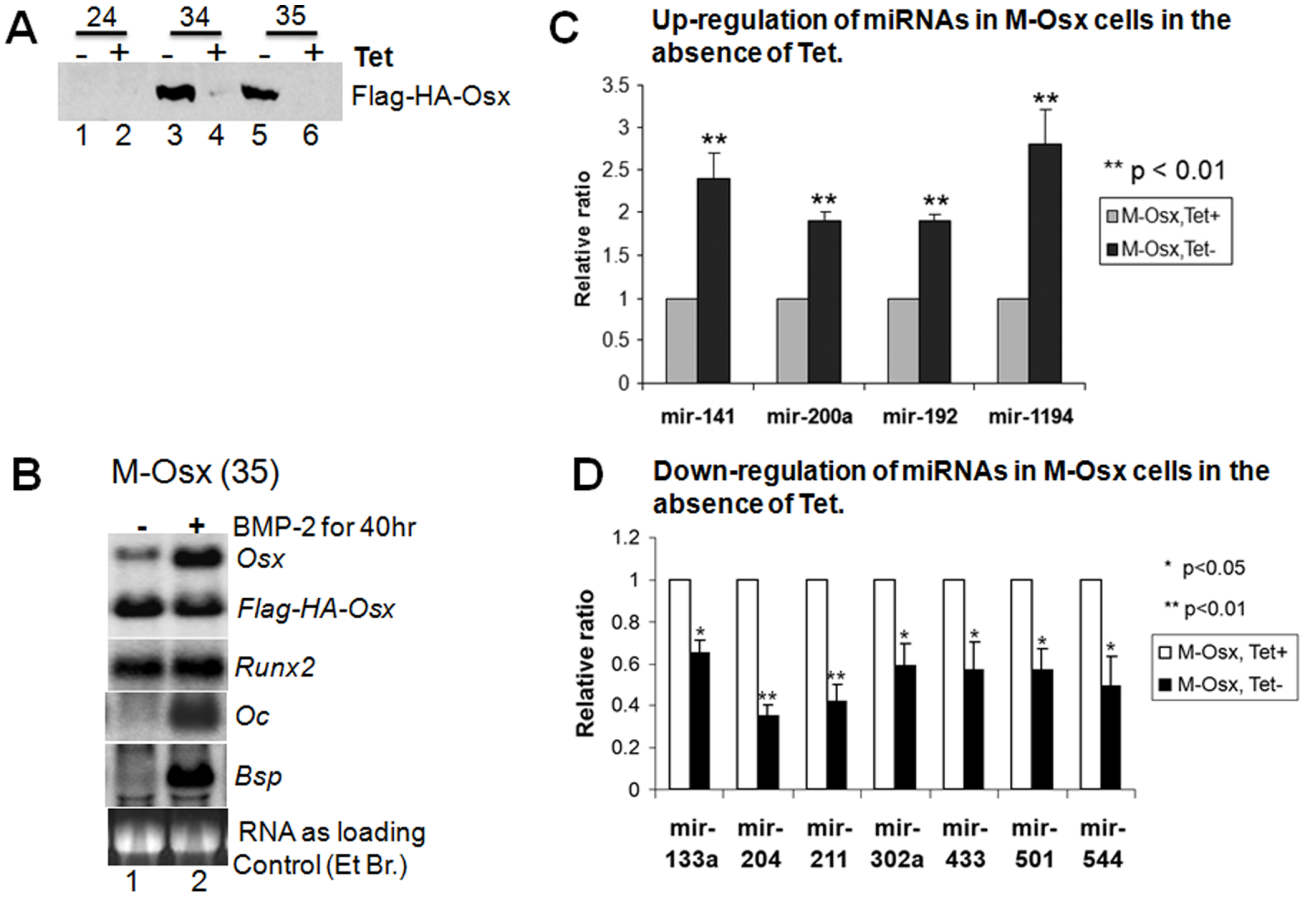
Generation of M-Osx cell line and expression of miRNAs in those cells. A: Western blot using anti-Flag antibody: lanes 1–6, cell lysates from clones 24, 34 and 35 in the absence or presence of Tet. **B:** Northern blot of RNAs from cells of clone 35 cultured without (lane 1) or with (lane 2) BMP2 for 40 h, hybridized with mouse *Osx*, *flag-HA*, mouse *Runx2*, mouse *Oc* and mouse *Bsp* cDNAs. **C, D:** Expression of miRNAs in M-Osx cells, which were cultured in the absence or presence of Tet for 48 h. The reported values are relative to expression of miRNAs in cells plus Tet (set as 1). All data are means ± S.D. (*n* = 3). *p<0.05; **p<0.01.

To further test whether expression of miRNAs listed in Tables 1 and 2 is regulated by Osx expression during osteogenesis, we engineered mouse preosteoblast MC3T3 cells by inducible expression of Flag-HA-tagged Osx (Flag-HA-Osx), which we named M-Osx. The positive clones were selected by G418 and hygromycin, and expression of the Flag-HA-Osx was verified by Western blot using anti-Flag antibody. As shown in Figure 1A, Flag-HA-Osx was highly expressed in positive clones number 34 and 35 in the absence of tetracycline (Tet) (Fig. 1A, lanes 3 and 5), but was not expressed in the presence of Tet (Fig. 1A, lanes 4 and 6). As a control, Flag-HA-Osx was not expressed in clone number 24 in the presence or absence of Tet (Fig. 1A, lanes 1 and 2). Therefore, cells from either clone 34 or 35 were expanded and maintained in the presence of Tet as the M-Osx cell line. To test whether M-Osx cells express the osteoblast differentiation markers *Bsp* and *Oc* under BMP2 treatment, cells from positive clone number 35 were cultured in the absence of Tet with or without BMP2 (Fig. 1B). After 40 h, cells without BMP2 treatment expressed a low level of endogenous *Osx*, but high levels of exogenous *Flag-HA-Osx* and endogenous *Runx2* (Fig. 1B, lane 1). However, expression of the osteoblast differentiation markers *Bsp* and *Oc* was still undetectable in those cells (Fig. 1B, lane 1). It suggests that when MC3T3 cells are cultured in the absence of BMP2 for 40 h, over-expression of Osx is not sufficient to turn on expression of its target genes, *Bsp* and *Oc*. In contrast, after cells were treated with BMP2, expression of *Bsp* and *Oc* was markedly increased, meanwhile, the levels of endogenous *Osx* and *Runx2* were also higher than those in cells without BMP2 (Fig. 1B, lane 2). By comparison, expression of *Flag-HA-Osx* was not affected by the BMP2 treatment (Fig. 1B). These results indicate that M-Osx cells have the capacity to express the osteoblast differentiation markers *Bsp* and *Oc* in response to BMP2 treatment.

### MiRNAs Positively Regulated by Osx Expression

In mouse calvarial tissue, after qPCR validation we found that expression of mir-141, -192, -200a, and -1194 was significantly down-regulated in the *Osx^−/−^* calvaria compared to their levels in WT (Table 1), indicating a tight positive correlation in expression between Osx and these miRNAs. To further confirm this correlation, the aforementioned M-Osx cells were cultured in the absence or presence of Tet for 48 h. We found that expression of mir-141, -192, -200a, and -1194 was significantly up-regulated in the non-Tet cells (Fig. 1C; p<0.01), which express a high level of *Flag-HA-Osx* (Fig. 1A, lane 5; Fig. 1B, lane 1). These results suggest that at the onset of preosteoblast differentiation, over-expression of Osx may positively regulate (either directly or indirectly) transcription of these miRNAs. Mir-141 and -200a are homologous. Since mir-141/200a has been known as Dlx5-targeting miRNAs [28], these findings also imply that Osx may negatively regulate Dlx5 protein synthesis via, at least in part, up-regulation of mir-141/200a. However, the role of mir-192 and mir-1194 in osteogenesis is yet to be known.

### MiRNAs Negatively Regulated by Osx Expression

As shown in Table 2, expression of mir-133a, -204, -211, -302a, -433, -501, and -544 was significantly up-regulated in the *Osx^−/−^* calvaria compared to their levels in WT littermates, indicating a strong negative correlation between Osx expression and expression of this group of miRNAs. To further confirm this negative correlation, we used qPCR to measure expression of these miRNAs in the aforementioned M-Osx cells cultured with or without Tet. Mir-204 and -211 are homologous. A recent study indicates that mir-133a and -204/211 are moderately expressed in mouse preosteoblastic MC3T3 cells [30]. When M-Osx cells were cultured for 48 h in the presence of Tet, which turns off expression of Flag-HA-Osx (Fig. 1A), we noticed that mir-133a, -204/211, along with mir-302a, -433, -501 and -544 were all expressed in these cells. However, when M-Osx cells were cultured for 48 h in the absence of Tet to turn on the high level expression of Flag-HA-Osx (Fig. 1A), expression of these miRNAs was significantly down-regulated (Fig. 1D, p<0.05 or 0.01). These results suggest that during the early-stage differentiation of preosteoblasts, over-expression of Osx may negatively regulate (either directly or indirectly) transcription of this group of miRNAs. Since mir-133a and -204/211 are known as Runx2-targeting miRNAs [29], [30], these observations also imply that Osx may modulate the cellular level of Runx2 protein via in part down-regulation of mir-133a and -204/211.

### Expression of mir-302a and -544 was not Sufficient to Inhibit BMP2-induced Alkaline Phosphatase Activity

**Figure 2:**
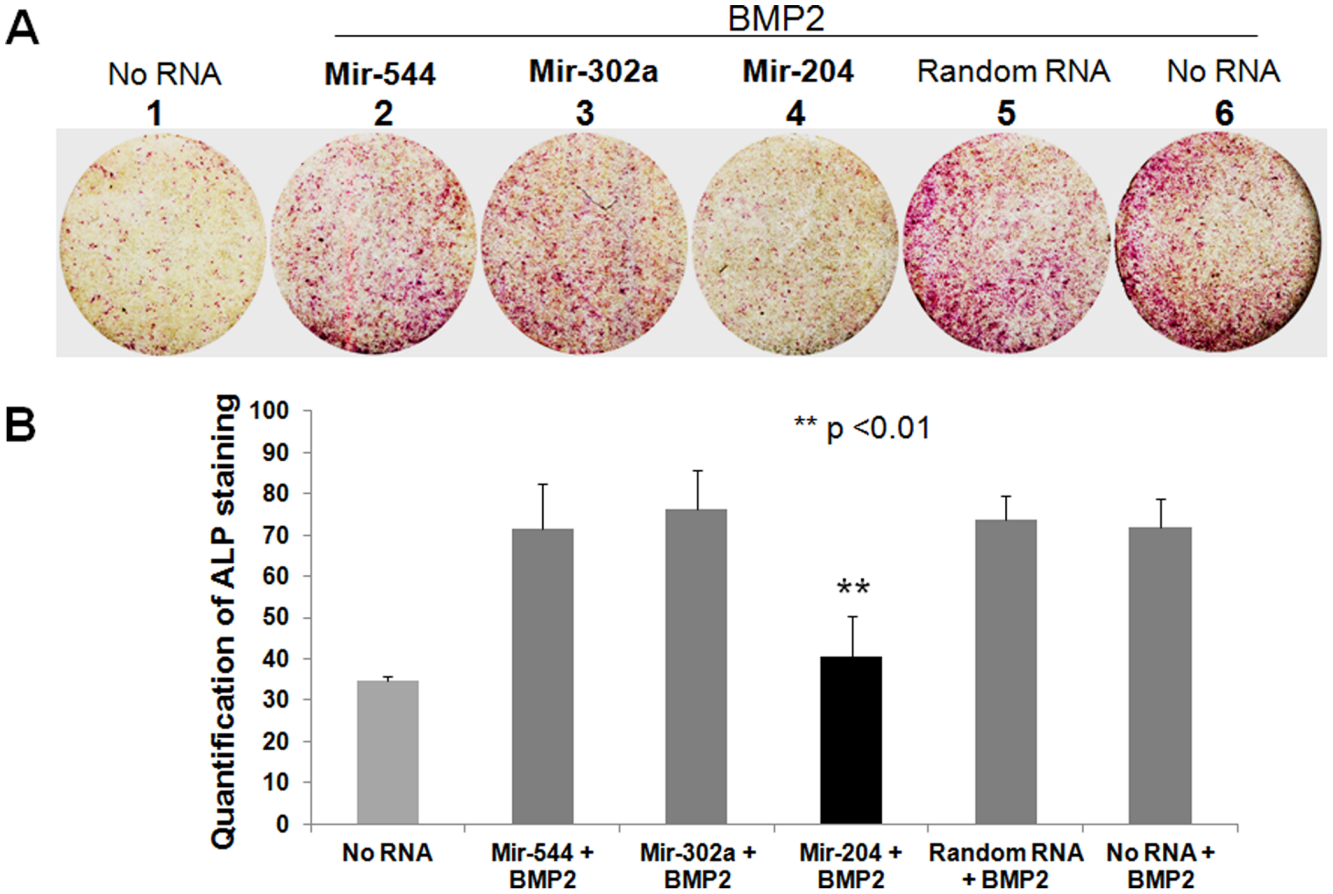
Expression of mir-302a or -544 in BMP2-treated C2C12 cells is not sufficient to inhibit ALP production. A: C2C12 cells were transfected with control RNA and different miRNAs, as shown in the panel. At 24h post-transfection, cells were treated with BMP2 as indicated**.** At 48 h after BMP2 treatment, cells were examined for ALP staining. **B:** Quantification of the ALP staining results in A.

Among the miRNAs that were negatively correlated with Osx expression (Table 2; Fig. 1D), mir-302a, -433, -501, and -544 have not been reported to be involved in osteogenesis. Using miRDB, an online database (http://mirdb.org/miRDB/index.html) for analysis of miRNA targets, we noticed that mir-302a might target the 3′-UTR of *Tgfbr2* and *Smad2*, whereas mir-544 might target the 3′-UTR of the *Bmpr2* and *Smad4/9* genes, suggesting that they might be involved in the TGF-β/BMP signaling pathway. To test whether over-expression of mir-302a or -544 inhibits the BMP2-induced ALP production during osteoblast differentiation, mouse C2C12 cells were used for RNA transfection and assay for ALP activity. We found that ALP activity was significantly reduced in mir-204-transfected cells treated with BMP2 for 72 h (Fig. 2A, compare well No.4 with wells No.5 and No.6; Fig. 2B), consistent with the previous study [27]. By comparison, there was no significant change in ALP staining in cells transfected with either mir-302a or -544 when compared to cells transfected with scrambled RNA or no RNA control (Fig. 2A, compare wells No.2 and No.3 with wells No.5 and No.6; Fig. 2B). These results indicate that over-expression of mir-302a or -544 in C2C12 cells, is not sufficient to repress the BMP2-induced ALP activity.

### Alkaline Phosphatase (ALP) is a Direct Target of mir-204

**Figure 3:**
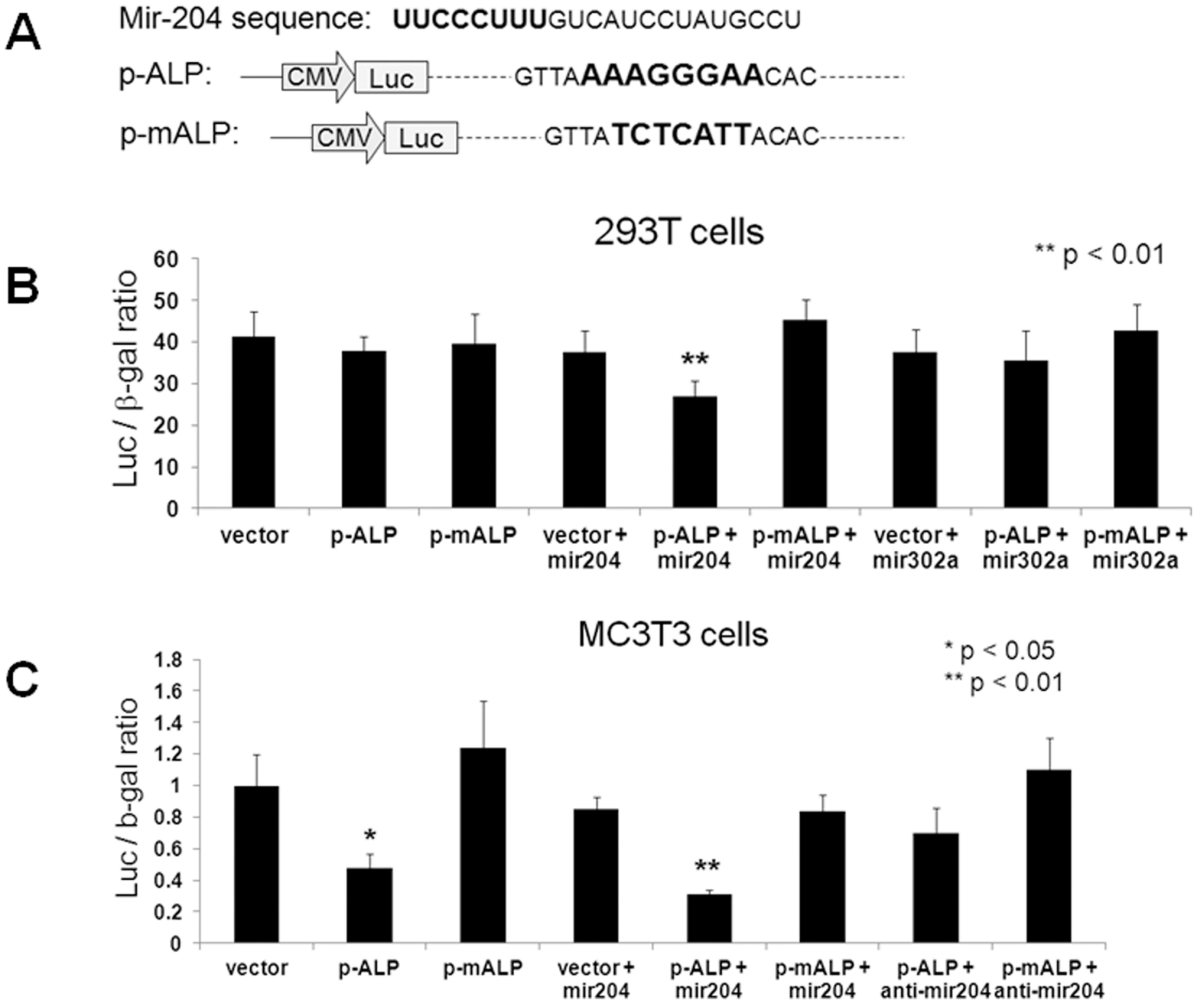
Expression of mir-204 inhibits *ALP* 3′-UTR reporter (p-ALP) activity. A: Schematic representation of wild-type and mutant *ALP* 3′-UTR reporter constructs with wild-type (p-ALP) or mutant (p-mALP) mir-204 seed sequence in the 3′-UTR of *ALP* gene. The mir-204 target sequence and its mutation in the 3′-UTR of the *ALP* gene were indicated in bold interface. **B:** Luciferase assay in 293T cells after transfection with different reporters and miRNAs as indicated in the panels. Mir-302a served as a control. **C:** Luciferase assay in MC3T3 cells after transfection with empty vector, p-ALP or p-mALP reporter with or without mir-204 or mir-204 inhibitor. All data represent means ± S.D. (*n* = 3). Statistical significance (p<0.05) was obtained by comparing with control cells treated with empty vector, mir-302a or mir-204 inhibitor.

Runx2 is essential for bone formation and ALP production. Since over-expression of mir-204/211 in cultured MC3T3 or BMP2-treated C2C12 cells reduces Runx2 protein level and decreases the ALP activity, it has been suggested that inhibition of ALP by mir-204/211 is Runx2-dependent [29], [30]. To date, it is not clear whether these miRNAs can directly interfere with ALP protein synthesis. From our miRNA target analysis using miRDB, we found that *ALP* was a putative target of mir-204. To confirm this, we fused a ∼100-bp 3′-UTR segment of the *ALP* gene, which contains a mir-204 seed sequence, into the pMIR-REPORT vector to generate a wild-type *ALP* 3′-UTR reporter construct (p-ALP) for luciferase assay (Fig. 3A). The mutant reporter construct (p-mALP) was generated by modifying 7 nucleotides of the mir-204 seed sequence in the 3′-UTR of the *ALP* gene (Fig. 3A). 293T cells were first used for transfection with plasmid DNA of empty vector, p-ALP, or p-mALP, in the absence or presence of mir-204 or mir-302a (which served as a control). At 48 h after transfection, we found that co-transfection of mir-204 with empty vector did not alter its luciferase activity, whereas co-transfection of mir-204 with wild-type p-ALP significantly reduced its luciferase activity (Fig. 3B; p<0.01). By comparison, control mir-302a, which presumably does not target *ALP* gene, did not affect the activity of p-ALP (Fig. 3B). In addition, co-transfection of either mir-204 or -302a with the mutant p-mALP reporter did not inhibit its luciferase activity (Fig. 3B). These results suggest that mir-204 targets the 3′-UTR of *ALP* gene. To further confirm these observations in osteogenic cell line, we then transfected MC3T3 cells with the empty vector, p-ALP or p-mALP, in the presence or absence of mir-204 or mir-204 inhibitor. After 48 h, we noticed a significant down-regulation of luciferase activity when expressing p-ALP, but not empty vector or p-mALP alone, in MC3T3 cells (Fig. 3C, p<0.05), implicating an inhibitory effect of endogenous mir-204 on p-ALP. Co-transfection of p-ALP with mir-204, but not mir-204 inhibitor, further decreased its luciferase activity (Fig. 3C, p<0.01). By comparison, co-transfection of p-mALP with mir-204 or mir-204 inhibitor did not significantly affect its luciferase activity (Fig. 3C). These results indicate that mir-204 directly targets the 3′-UTR of *ALP* gene. The direct interaction between mir-204 and *ALP* gene may contribute to the mir-204-induced inhibition of osteoblast differentiation in MC3T3 as well as BMP2-treated C2C12 cells.

### *Sost,* which Encodes a Wnt Signaling Antagonist, is also a Direct Target of mir-204

**Figure 4:**
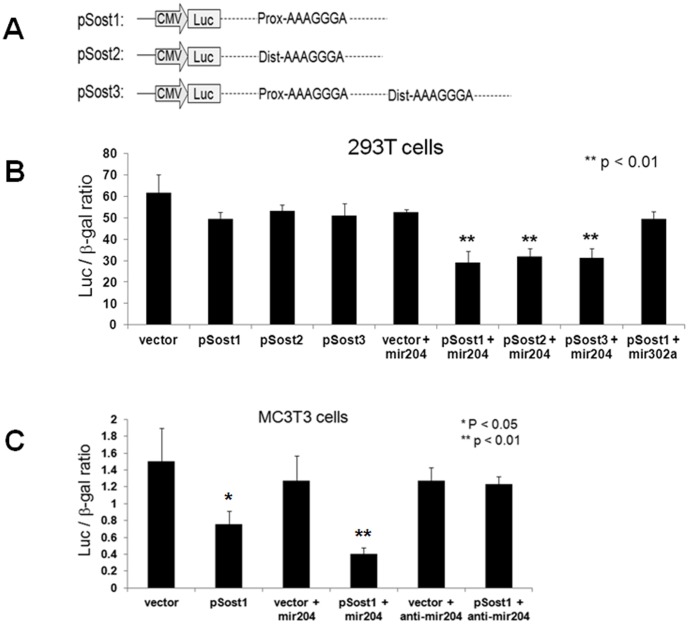
Expression of mir-204 inhibits *Sost* 3′-UTR reporter (pSost1-3) activities. A: Schematic representation of three *Sost* 3′-UTR reporter constructs containing proximal (pSost1) or distal (pSost2) or both (pSost3) mir-204 seed sequences in the 3′-UTR of *Sost* gene. **B:** Luciferase assay in 293T cells after transfection with reporter constructs and miRNAs as indicated in each panel. Mir-302a served as a control. **C:** Luciferase assay in MC3T3 cells after transfection with empty vector or pSost1 reporter in the absence or presence of mir-204 or mir-204 inhibitor.

The Wnt/β-catenin signaling pathway plays a major role in osteoblast proliferation and differentiation [19], [20], [21], [42], [43]. Since the *Sost* gene which encodes Sclerostin, an important antagonist of the Wnt signaling, was listed among the predicted targets of mir-204, we wanted to investigate whether the 3′-UTR of *Sost* is also a direct target of mir-204. Three PCR fragments, containing the mir-204 seed sequence in either the proximal or distal or both regions of the *Sost* 3′-UTR, were fused to the pMIR-REPORT vector to generate three *Sost* 3′-UTR reporter constructs, pSost1, pSost2 and pSost3, for luciferase assays (Fig. 4A). Empty-vector or each *Sost* 3′-UTR reporter plasmid was transfected with or without mir-204 into the cultured 293T cells. At 48 h after transfection, we did not observe significant change in luciferase activity when expression of empty vector or each reporter in the absence of mir-204 (Fig. 4B). Co-transfection of mir-204 with empty vector did not affect its luciferase activity. However, co-expression of mir-204 with any of those three reporters significantly decreased their luciferase activity (Fig. 4B; p<0.01). By comparison, co-transfection of control mir-302a with pSost1 did not affect its activity. These observations suggest that the 3′-UTR of *Sost* is a direct target of mir-204. To confirm these results in osteoblastic cell line, we transfected the MC3T3 cells with empty vector or pSost1 reporter in the presence or absence of mir-204 or mir-204 inhibitor. After 48 h, we observed a significant reduction in luciferase activity of pSost1 reporter alone when compared to empty vector alone (Fig. 4C; p<0.05), suggesting an inhibitory effect of endogenous mir-204 on pSost1 reporter. Co-transfection of pSost1 with mir-204 further decreased the reporter activity (Fig. 4C; p<0.01). In contrast, co-transfection of either empty vector or pSost1 with mir-204 inhibitor did not significantly alter their luciferase activity (Fig. 4C). These data demonstrate that mir-204 directly targets the 3′-UTR of *Sost* gene.

**Figure 5:**
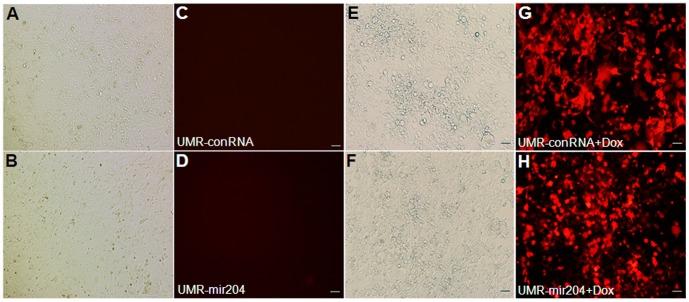
Generation of UMR-conRNA and UMR-mir204 stable cell lines. **A–D:** UMR-conRNA (A, C) and UMR-mir204 (B, D) cells were cultured in the absence of doxycycline (Dox). After 54 h, the cultured cells were examined under light microscope (A, B) and fluorescence microscope (C, D). **E–H:** The UMR-conRNA (E, G) and UMR-mir204 (F, H) cells were cultured in the presence of Dox. After 54 h, the cultured cells were examined under light microscope (E, F) and fluorescence microscope (G, H).

**Figure 6:**
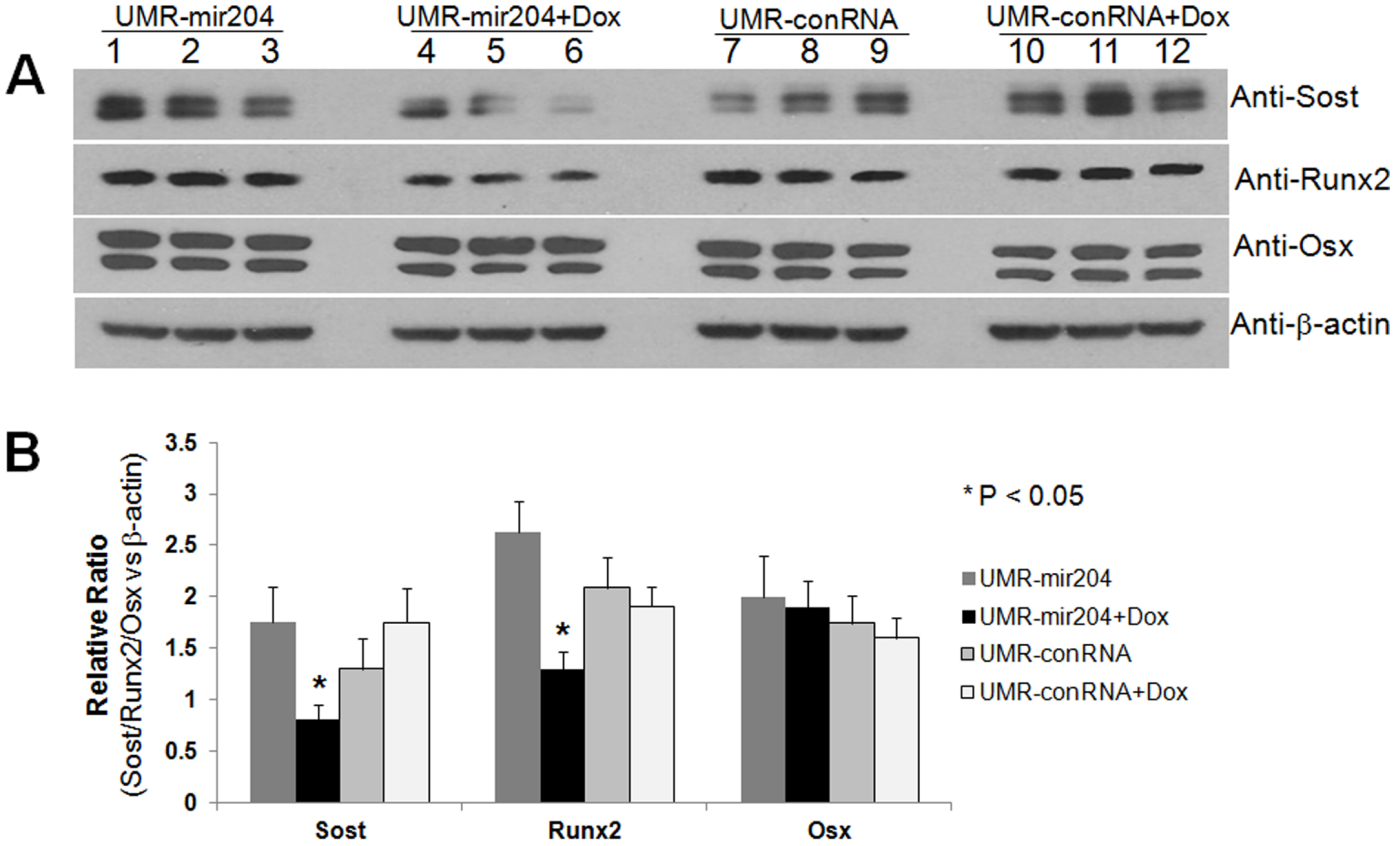
Expression of mir-204 in UMR cells attenuates Sost and Runx2, but not Osx protein. **A:** Western blots: The UMR-conRNA and UMR-mir204 cells were cultured in the absence or presence of Dox. After 54 h, the cultured cells were harvested for Western blots using antibody against Sost, Runx2, Osx or β-actin. **B:** Quantification of Western blot results in A.

All data represent means ± S.D. (*n* = 3). Statistical significance (p<0.05) was obtained by comparison with control cells treated with empty vector, mir-302a or mir-204 inhibitor.further examine whether expression of mir-204 directly attenuates Sost protein in osteoblasts, we used the osteogenic UMR-106 cells for this study. The UMR-106 cell line is a clonal derivative of a transplantable rat osteosarcoma and shares several phenotypic features of mature osteoblasts. Therefore, Runx2, Osx and Sost are simultaneously expressed in these cells [13]. The UMR-106 cells were first engineered by stably expressing non-silencing control RNA (conRNA) or mir-204 plasmid vector to generate two stable cell lines, UMR-conRNA and UMR-mir204. In both cell lines, expression of conRNA or mir-204, followed by expression of Red Fluorescent Protein (RFP), is inducible. As shown in Figure 5, in the absence of doxycycline (Dox) expression of conRNA or mir-204 was off, therefore, no red color was seen under fluorescence microscope (Fig. 5C, D). However, when cells were treated with Dox, expression of conRNA or mir-204 was on, and subsequently triggered expression of RFP, which shows red color under fluorescence microscope (Fig. 5G, H). To test changes in levels of Sost, Runx2 and Osx proteins, the UMR-conRNA and UMR-mir204 cells were cultured in DMEM supplemented with 10% FBS in the presence or absence of Dox. After 54 h, the levels of Sost and Runx2 proteins were significantly decreased in the UMR-mir204 cells treated with Dox (Fig. 6A, lanes 4–6 in rows 1 and 2; Fig. 6B), whereas the level of Osx protein was not significantly altered in those cells (Fig. 6A, lanes 4–6 in row 3; Fig. 6B). By comparison, there was no significant change in levels of Sost, Runx2 and Osx proteins in UMR-mir204 cells without Dox (Fig. 6A, lanes 1–3; Fig. 6B) or UMR-conRNA cells treated with Dox (Fig. 6A, lanes 10–12; Fig. 6B). These results suggest that mir-204 directly attenuates the Sost protein synthesis. Since expression of mir-204 is negatively regulated by Osx expression, these results also imply that repression of mir-204 by Osx may contribute, at least in part, to the Osx-mediated stimulation of the *Sost* gene product.

## Discussion

In this study, we have identified groups of miRNAs which are differentially expressed in calvaria of the E18.5 *Osx^−/−^* embryos compared to wild type embryos, and verified the correlation between the levels of these miRNAs and Osx expression in cultured cells. Among those miRNAs differentially regulated by Osx, mir-133a and -204/211, which target Runx2, was up-regulated in *Osx^−/−^* calvaria and down-regulated in M-Osx cells when Osx was over-expressed. One hypothesis suggested by our in vivo data is that mir-133a and -204/211 may be part of a physiological feedback system that coordinates the levels of Osx and Runx2 protein in osteoblasts to optimize the transcriptional efficiency of these two transcription factors at their target gene promoters. Indeed, Runx2 and Osx have common target genes in osteoblasts [15]. Since we know that the level of Runx2 mRNA increases in *Osx^−/−^* calvaria [14], we speculate that mir-133a and -204/211 might attenuate the effect of this increase.

Previous studies showed that expression of mir-133a and -204/211 was down-regulated in BMP2-treated C2C12 cells, but up-regulated in adipocytes [27], [29]. Since *Osx* expression is induced in BMP2-treated C2C12 cells, and there is no *Osx* expression in adipocytes, these previous studies suggest an inverse correlation between *Osx* expression and expression of mir-133a and -204/211. The up-regulation of these miRNAs in Osx^−/−^ calvaria in vivo is not inconsistent with the results in C2C12 cells in vitro. However, in another in vitro cell culture study using MC3T3 cells, no difference in the levels of mir-133a and -204/211 transcripts was seen at the early stage of cell differentiation, whereas their levels were increasing at the later stages [30]. Given that each of the experimental system is different, it is challenging to provide a coherent explanation that would reconcile the different results.

**Figure 7:**
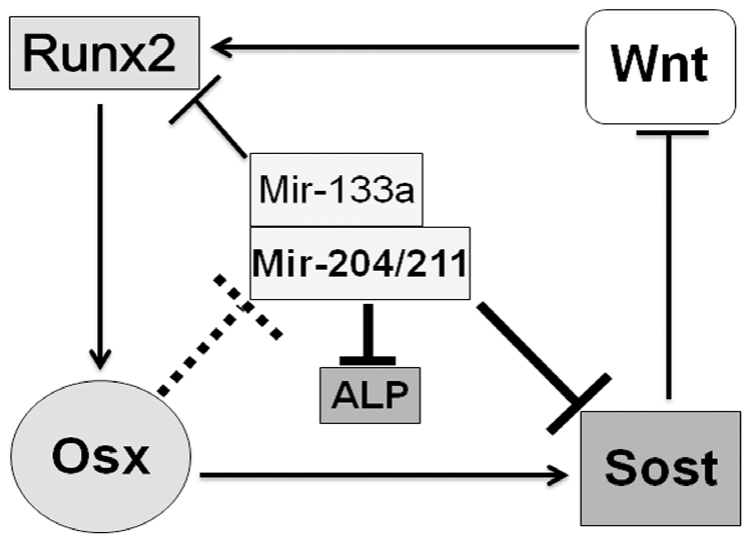
Schematic model for depicting a role of Osx in regulation of Runx2, Sclerostin and ALP through inhibition of miRNA expression. The solid lines with bars show inhibition; the dashed lines with bars show inhibition with unclear mechanisms; the arrow represents activation; the new findings of this study are shown in thicker lines, whereas the known information is shown in thinner lines.

Expression of *Runx2* and *Osx* is regulated by a broad signaling network including members of the Wnt family. The canonical Wnt signaling pathway plays a crucial role in regulation of osteoblast proliferation, osteoclast differentiation and bone remodeling. Our previous experiments have suggested that Osx stimulates expression of the genes for two Wnt signaling antagonists, DKK1 and Sclerostin. The implication of these results is that Osx would promote osteoclast differentiation and hence bone remodeling. Our present experiments suggest that mir-204/211 can target the 3′-UTR of *Sost* gene and attenuate its protein synthesis. Given that in the absence of Osx in vivo the levels of this miRNA in calvaria increases, and that in the cultured M-Osx cells, increased level of Osx reduces the level of this miRNA transcript, we hypothesize that mir-204/211 constitutes a second layer of control of canonical Wnt signaling that reinforce the transcriptional control of *Sost* by Osx in osteogenesis (Fig. 7).

ALP is an early marker of osteoblast differentiation. Since both *Runx2^−/−^* and *Osx* ^−/−^ mice showed little or no expression of ALP in osteoprogenitors [2], [3], [4], it indicates that ALP is an important downstream marker of Runx2 and Osx. Therefore, when over-expressing the Runx2-targeting mir-204/211 in cultured MC3T3 or BMP2-treated C2C12 cells [29], [30], (well 4 in Fig. 2), inhibition of ALP has been thought to be dependent upon attenuation of Runx2 protein by these miRNAs. Here, we provide in vitro evidence showing that mir-204/211 can also directly target the 3′-UTR of *ALP* (Fig. 3). Since *Osx* expression in cultured MC3T3 or BMP2-treated C2C12 cells is often induced concurrent with osteoblast differentiation and ALP production [28], the negative regulation of mir-204/211 by Osx implies that Osx may play a role in maintaining ALP protein level via in part down-regulation of mir-204/211 (Fig. 7).

Mir-302a and -544 have not been reported to be involved in osteogenesis. Target analysis showed that mir-302a might target the 3′-UTR of *Tgfbr2* and *Smad2*, whereas mir-544 might target the 3′-UTR of *Bmpr2* and *Smad4/9* genes. Although we did not test this target prediction, our finding that over-expression of either mir-302a or -544 was unable to modulate the BMP2-induced osteoblast differentiation marked by ALP production (Fig. 2), implies that the inhibition of target genes by mir-302a or -544, if any, may be compensated by other members of Smad family or *Bmpr1*. In addition, our study shows that Osx can up-regulate expression of mir-141/200a. Since mir-141/200a directly targets *Dlx5*, we hypothesize that during osteogenesis Osx may have the ability to prevent excessive production of Dlx5 protein via in part up-regulation of mir-141/200a. However, a previous study showed when MC3T3 cells were treated with BMP2, expression of Dlx5 along with Osx was increased, whereas expression of the endogenous mir-141/200a was down-regulated [28], implicating a negative correlation between Osx and expression of mir-141/200a. This seems inconsistent with our current finding. It is likely that exogenous BMP2 in cultured cells may inhibit the transcription of mir-141/200a.

Taken together, our data show a tight correlation between the master osteogenic transcription factor Osx and miRNAs involved in bone formation. It will be interesting to know how Osx coordinately modulates, either directly or indirectly, expression of these miRNAs to maintain an appropriate level of Runx2, Sclerostin, ALP and Dlx5 proteins for optimal differentiation and function of osteoblasts.

## Supporting Information

Figure S1 **A representative heat map.** This heat map shows expression of the selected miRNAs (listed in Tables S1 and S2) in each miRNA chip hybridized with calvarial RNAs of three individual wild type (WT, A597.13, A548.11, A600.17) and three individual Osx^−/−^ (KO, A599.15, A596.12, A598.14) mouse embryos at E18.5.
(TIF)

Table S1 **Up-regulation of miRNAs in miRNA array chips.**
(XLS)

Table S2 **Down-regulation of miRNAs in miRNA array chips.**
(XLS)

Table S3 **Forward primer sequences for qPCR assays.**
(XLS)
