# Supplementary figures and images for: Identification and Characterization of MicroRNAs Controlled by the Osteoblast-Specific Transcription Factor Osterix
Source: PLoS One. 2013 Mar 5;8(3):e58104. doi: 10.1371/journal.pone.0058104 (PMC3589352; doi:10.1371/journal.pone.0058104)

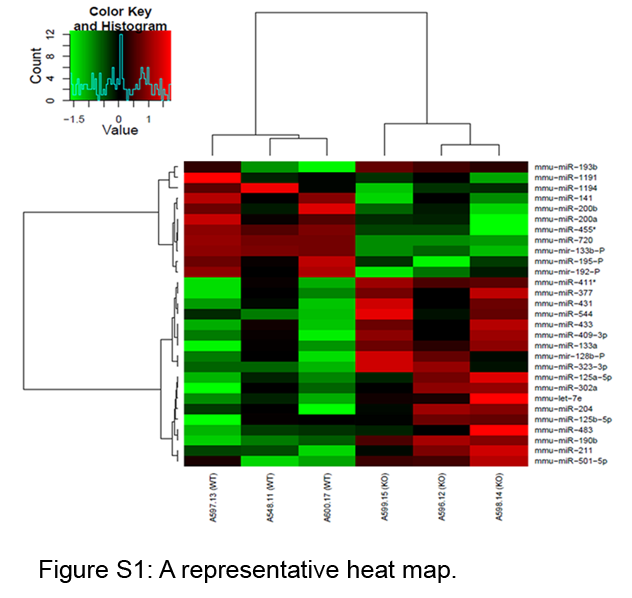

Supplement: Figure S1 — A representative heat map. This heat map shows expression of the selected miRNAs (listed in Tables S1 and S2) in each miRNA chip hybridized with calvarial RNAs of three individual wild type (WT, A597.13, A548.11, A600.17) and three individual Osx−/− (KO, A599.15, A596.12, A598.14) mouse embryos at E18.5. (TIF) [file pone.0058104.s001.tif]
